# Outcome of Facial Nerve Integrity After Parotid Gland Surgery With and Without Intraoperative Monitoring: A Ten-Year Retrospective Study

**DOI:** 10.3390/jcm14041156

**Published:** 2025-02-11

**Authors:** Giulio Cirignaco, Gabriele Monarchi, Enrico Betti, Mariagrazia Paglianiti, Lisa Catarzi, Alessandro Tel, Luigi Angelo Vaira, Paolo Balercia, Giuseppe Consorti

**Affiliations:** 1 Department of Medicine, Section of Maxillo-Facial Surgery, University of Siena, Viale Bracci, 53100 Siena, Italy; g.cirignaco@student.unisi.it (G.C.); gabriele.monarchi@gmail.com (G.M.); mgpaglianiti@gmail.com (M.P.); lisa.catarzi@gmail.com (L.C.); 2 Division of Maxillofacial Surgery, Department of Neurological Sciences, Marche University Hospitals-Umberto I, 60126 Ancona, Italy; enrico.betti@ospedaliriuniti.marche.it (E.B.); paolo.balercia@ospedaliriuniti.marche.it (P.B.); 3 Clinic of Maxillofacial Surgery, Head-Neck and NeuroScience Department, University Hospital of Udine, 33100 Udine, Italy; alessandro.tel@asufc.sanita.fvg.it; 4 Maxillofacial Surgery Operative Unit, Department of Medicine, Surgery and Pharmacy, University of Sassari, Viale San Pietro 43/B, 07100 Sassari, Italy; lavaira@uniss.it; 5 Biomedical Science Department, PhD School of Biomedical Science, University of Sassari, 07100 Sassari, Italy

**Keywords:** facial nerve, parotid gland surgery, benign parotid tumor, intraoperative nerve monitoring, postoperative facial nerve injury

## Abstract

**Background**: Facial nerve injury is one of the most concerning complications of parotid gland surgery, with temporary and permanent dysfunction rates varying widely in the literature. This study aimed to identify factors associated with facial nerve injury during surgery for benign parotid tumors and assess the protective efficacy of intraoperative nerve monitoring (NIM) in preventing nerve injury. **Methods**: This retrospective study analyzed 329 patients who underwent parotid gland surgery between 2010 and 2023. Data collected included patient demographics, tumor characteristics (size, location, histology), surgical parameters (operation time, NIM usage), and postoperative nerve function evaluated using a modified House–Brackmann scale. Descriptive and inferential statistical analyses, including Chi-square tests and logistic regression, were employed to identify predictors of facial nerve outcomes. **Results**: Facial nerve injury occurred in 5.2% of patients, comprising 4.6% temporary and 0.6% permanent damage. Tumors located in the deep and inferior lobes significantly increased the risk of facial paralysis/palsy (*p* = 0.035), while tumor size and histology showed no significant associations. Using NIM significantly reduced the risk of facial nerve injury (*p* < 0.05; OR: 0.35, 95% CI: 0.25–0.50). Age was also identified as a significant predictor of nerve dysfunction (*p* < 0.05). **Conclusions**: The findings emphasize the importance of NIM in mitigating facial nerve injury, especially in anatomically complex tumor locations. Tailored surgical approaches based on tumor location and patient-specific factors, combined with the routine use of NIM, are recommended to optimize nerve preservation and improve postoperative outcomes.

## 1. Introduction

Facial nerve injury is a critical and potentially debilitating complication of parotid gland surgery, affecting both benign and malignant cases. Despite advancements in surgical techniques and perioperative care, the rates of temporary and permanent dysfunction remain significant, varying widely from 5% to 65% depending on factors such as tumor characteristics, patient anatomy, surgeon experience, and the use of intraoperative nerve monitoring systems (IONM) [1,2,3].

Surgical techniques for parotid tumors have evolved considerably over the last century. In the early 20th century, intracapsular enucleation was the standard for treating benign tumors, but due to residual capsular and tumor cells, it resulted in recurrence rates as high as 20–45% [4].

The adoption of superficial parotidectomy, utilizing anterograde or retrograde facial nerve dissection, significantly reduced recurrence rates to approximately 5%, thus leading to increased complications, including Frey’s syndrome, salivary fistula, and aesthetic concerns [4].

Approximately 80% of parotid tumors are benign, with pleomorphic adenoma being the most common, followed by Warthin tumors and rarer neoplasms such as basal cell tumors and oncocytoma [5].

The facial nerve, exiting the stylomastoid foramen and traversing the parotid gland in a complex branching pattern, is highly susceptible to injury during surgery, particularly in cases involving deep lobe tumors, larger sizes, or proximity to nerve branches. While malignant tumors inherently pose a higher risk of nerve injury, benign lesions also present significant challenges, such as encapsulation, recurrence, or scarring, necessitating meticulous surgical techniques for nerve preservation [3,6]. Although the facial nerve is entirely dissected during superficial parotidectomy, temporary paralysis can still occur in some cases [7].

To address these challenges, intraoperative nerve monitoring (IONM) has been increasingly adopted as a valuable adjunct in parotid gland surgery. Nerve monitoring is commonly used in thyroid surgery to preserve the function of the laryngeal nerve, whereas this does not apply to facial nerve monitoring. IONM provides real-time electrophysiological feedback, assisting surgeons in identifying and preserving the facial nerve and its branches throughout the procedure [5,7]. While studies report reduced nerve injury rates, its efficacy remains debated. Several studies have demonstrated the potential benefits of IONM, reporting a reduced incidence of transient and permanent nerve injury, particularly in cases with high anatomical complexity [2]. However, the utility of IONM is debated: while some studies emphasize its benefits [1], others report no significant impact on facial nerve outcomes, particularly when surgery is performed by experienced surgeons skilled in meticulous dissection techniques [7].

These conflicting findings underscore the need for robust, evidence-based research to clarify the role of IONM in modern parotid surgery. While previous studies have largely focused on isolated factors such as tumor location, size, or histology, comprehensive analyses of the interplay between these variables and their impact on postoperative nerve function remain limited [1,4].

To assess the facial nerve function, one of the most used and widely known scale is the House–Brackmann, which evaluates patients regarding facial appearance, symmetry, and tone at rest, as well as and movement of the forehead, eye, and mouth region [8].

This study aims to address these gaps by evaluating predictors of facial nerve injury following parotidectomy and assessing the impact of IONM on surgical outcomes. By analyzing tumor characteristics (location, size, histology), surgical factors (duration, complexity, revision status), and patient demographics (age, sex), we seek to clarify the efficacy of IONM and provide insights for optimizing surgical strategies and improving patient outcomes.

## 2. Materials and Methods

This retrospective study included 529 patients who underwent parotid tumor surgery at the Maxillo-Facial Surgery Unit of “Azienda Ospedaliera Ospedali Riuniti” in Ancona (Marche, Italy) between January 2010 and December 2023. Patient information (age, sex, tumor side, histological diagnosis, tumor size, and operation time) was retrieved from medical records and tabulated using Microsoft Excel (Version 16.63.1; Microsoft Corporation, Redmond, WA, USA). Tumor size was evaluated intraoperatively and confirmed via histopathological reports. Personal and sensitive data were excluded from the dataset to ensure confidentiality.

Inclusion criteria accounted for patients over 18 years old with a postoperative diagnosis of benign parotid tumor. Exclusion criteria comprised malignant parotid tumors, recurrent neoplasms, preoperative facial nerve deficits (House–Brackmann score), inflammatory episodes of the salivary gland, and combined surgeries involving the neck. Of the initial 529 patients, 329 met the inclusion criteria. Surgical timing was evaluated from the moment of incision to the last skin suture as documented in the hospital surgical log as the time required to set up the monitor equipment was not considered.

At the time of surgery, patients were assigned to either the NIM or no-NIM group randomly as part of the clinical practice in our institution. This assignment was independent of tumor characteristics, such as size, location, or proximity to the facial nerve. Random allocation was implemented to avoid selection bias and ensure comparability between the two groups. In this retrospective study, we analyzed these data to evaluate the impact of NIM on postoperative facial nerve outcomes.

In this study, we adopted a modified House–Brackmann score to mitigate the potential subjective bias of the observer documented in the patient medical records, grouping together scores III and IV, as well as V and VI, to minimize a possible bias for the subjective evaluation that led us to consider four levels of dysfunction, with level I meaning no dysfunction, level II mild dysfunction, level III moderate dysfunction, and level IV severe dysfunction [9].

The facial nerve function analysis was preoperatively assessed the day before surgery and postoperatively on the first day, the first month, the third month, and the sixth month. Levels II and III were classified as facial paralysis, representing mild to moderate dysfunction, while Level IV was categorized as facial palsy, indicating severe or complete dysfunction.

### 2.1. Intraoperative Monitoring and Operative Technique

In both patient groups, general anesthesia was administered following the standard protocols established in our clinical practice. Anesthesia induction was carried out with an intravenous bolus of propofol (2 mg/kg) combined with a reduced dose of rocuronium (0.3 mg/kg). Continuous infusion of remifentanil, at a rate of up to 1 mcg/kg/min, ensured appropriate analgesia.

As curare was chosen for anesthesia induction at a dosage of 1 mg/kg, sugammadex (2 mg/kg) was administered once the surgery started and the parotid gland was reached. Analgesia was maintained through continuous remifentanil infusion at doses ranging between 0.05 and 0.2 μg/kg/min.

All first and second surgeons performed the procedures using 3.5 magnifying surgical loupes. The preference for working distance varied slightly among operators, ranging from 35 to 40 mm. No surgeries were conducted without the use of magnifying loupes, irrespective of whether the NIM was utilized or not.

The operative setup was standardized to minimize interindividual variability. The first surgeon was seated on the same side as the affected parotid gland, ensuring optimal ergonomic access and visibility. The operating table was slightly rotated toward the contralateral side to facilitate the approach for the assistant surgeon, who remained standing on the opposite side. A third assistant, also standing, provided additional support as needed. This setup was uniformly applied for both right and left parotid gland surgeries.

The device used for intraoperative monitoring was the Nerve Integrity Monitor (NIM^®^) (Medtronic, Minneapolis, MN, USA). The system offers audio-visual feedback based on electromyographic (EMG) signals produced during intraoperative nerve stimulation. It consists of two primary components: a recording electrode and a nerve stimulation probe, which may be monopolar or bipolar, and is connected to a pulse generator. The recording electrode is designed to analyze a variable volume of tissue, while a grounding electrode—typically placed on the patient’s shoulder near the monitor or on the sternum—minimizes electrical interference.

Four recording electrodes are employed: one positioned on the lower forehead for the frontal muscle, one on the infraorbital region for the Orbicularis oculi muscle (temporal and zygomatic nerve), one on the upper lip for the Orbicularis oris muscle (buccal nerve), and one on the lower lip for the mental muscle. These electrodes are inserted subcutaneously into the respective muscles after cleansing the area with alcohol wipes.

Stimulation intensity was adjusted (1.5–2 mA for mapping, 0.5–0.8 mA for detailed dissection). At the end of the procedure, the NIM^®^ generated a PDF report with EMG recordings [7,10].

### 2.2. Postoperative Management

A compression dressing was applied immediately post-surgery and replaced daily. Routine antibiotic prophylaxis was not administered unless clinically indicated. Daily evaluations included clinical assessments and sonography for fluid collections (e.g., seromas or hematomas). If fluid collections were identified, local drainage and antiseptic treatment were performed. Anti-inflammatory drugs or corticosteroids were used as needed, with systemic antibiotics reserved for specific indications [11].

### 2.3. Statistical Analysis

The dataset, comprising 329 cases, was analyzed to identify significant predictors and associations between clinical parameters and the occurrence of facial paralysis or palsy. Descriptive statistics summarized demographic and clinical characteristics, while inferential methods assessed the relationships between key variables. Categorical variables were analyzed using Chi-square tests, and continuous variables were evaluated with *t*-tests or non-parametric equivalents as appropriate. Logistic regression identified independent predictors, with adjusted odds ratios (ORs) and 95% confidence intervals (CIs) reported for significant variables. Statistical significance was set at *p* < 0.05. Analyses were conducted using Jamovi Statistics (Version 2.6.17.0; The Jamovi Project, Sydney, Australia) 

## 3. Results

### 3.1. Descriptive Statistics

#### 3.1.1. Demographic and Clinical Characteristics

The dataset included a total of 329 patients undergoing parotid gland surgery. Demographic analysis revealed a balanced sex distribution, with 174 females (52.9%) and 155 males (47.1%). The surgeries were nearly equally distributed between the right side (167 cases, 50.8%) and the left side (162 cases, 49.2%).

The mean age of the patients was 61.3 years (SD = 15.2), with a range from 20 to 94 years. The age distribution showed that 25% of patients were under 52 years, the median age was 61 years, and 25% were over 71 years.

#### 3.1.2. Tumor Characteristics

The mean tumor size was 3.1 cm (SD = 0.7), ranging from 0.6 to 7.6 cm. The 25th percentile was 2.7 cm, the median size was 3.0 cm, and the 75th percentile was 3.5 cm.

Tumor histology was predominantly pleomorphic adenoma, which accounted for 171 cases (52.0%), followed by Warthin’s tumor with 137 cases (41.6%). Less common histological types included lymphoepithelial cysts (15 cases, 4.6%) and oncocytomas (5 cases, 1.5%).

Regarding tumor location, most were in the superficial lobe (238 cases, 72.3%), with fewer in the inferior lobe (75 cases, 22.8%) and the deep lobe (16 cases, 4.9%) (Table 1).

#### 3.1.3. Surgical Outcomes

The mean operation time was 107.2 min (SD = 34.2), ranging from 23 to 180 min. The median was 110 min, with 25% of surgeries lasting under 90 min and 25% exceeding 128 min.

NIM was used in 135 surgeries (41.0%), while 194 (59.0%) were performed without it. Postoperative facial nerve dysfunction was observed in 17 cases (5.2%). Injuries were more frequent in the temporal-zygomatic branch (64.7%), followed by the marginalis branch (23.5%) and combined lesions (11.8%). Facial nerve dysfunction was observed in 7 cases (41.2%) with NIM and in 10 cases (58.8%) without NIM.

All data are presented in Table 1.

**Table 1:** Descriptive patient data.

		NIM	No NIM	Total
Sex	Male	63	72	155
	Female	72	102	174
Mean age (Years)		60.1	62.2	61.3
Tumor site	Superficial lobe	95	143	238
	Inferior lobe	30	45	75
	Deep lobe	10	6	16
Histopathology	Pleomorphic adenoma	70	101	171
	Warthin’s tumor	50	87	137
	Lymphoepithelial cyst	10	5	15
	Oncocytomas	5	0	5
Operative Time (Min)		105.3	108.5	107.2
Facial nerve dysfunction	Total cases	7	10	17

### 3.2. Inferential Statistical Analysis

The Chi-square test (χ^2^) was used to determine the association between categorical variables, such as sex, tumor site, and the use of the nerve integrity monitor (NIM), with the occurrence of facial nerve injury.

- Sex: no significant association was observed between patient sex and the occurrence of facial paralysis or palsy (*p* = 0.451), indicating that likelihood is not influenced by the patient’s sex.
- Side of the tumor: the side of the tumor (left or right) did not show a significant relationship with paralysis or palsy (*p* = 0.574), suggesting no dependency between tumor side and facial nerve integrity outcomes.
- Histological Type: Tumor histology was not significantly associated with the risk of paralysis or palsy (*p* = 0.317). This implies that the histological type of the tumor does not predict the likelihood of nerve damage.
- Tumor location (superficial or inferior) was significantly associated with paralysis or palsy (*p* = 0.035). Patients with tumors in the inferior lobe showed a higher incidence of facial nerve injury compared to those with tumors in the superficial lobe. This indicates that tumor location plays an important role in predicting the risk of damage, potentially due to proximity to the main facial nerve.

In the Chi-square test, the association between NIM usage and the risk of facial injury was not statistically significant (*p* = 0.229). However, in logistic regression analysis, after adjusting for other variables, NIM usage showed a significant protective effect against paralysis or palsy (*p* < 0.05).

#### 3.2.1. Comparison of Means (*T*-Tests)

The independent samples *t*-test was employed to compare the means of continuous variables (such as age, tumor size, and operation time) between patients with and without facial nerve injury.

- Age: Patients who developed facial paralysis or palsy had a significantly higher mean age compared to those who did not (t = −2.41, *p* = 0.027 95% CI for mean difference: 3.2 to 9.5 years). This result indicates that age is a significant factor, with older patients being at higher risk.
- Tumor size: There was no significant difference in tumor size between patients with and without facial paralysis or palsy (t = 1.30, *p* = 0.211 95% CI for mean difference: −0.2 to 0.6 cm). This suggests that tumor size alone does not influence the likelihood of nerve injury.
- Operation time: Surgical duration was not significantly associated with the occurrence of paralysis or palsy (t = 1.60, *p* = 0.126 95% CI for mean difference: −5.0 to 12.5 min). This implies that longer surgeries do not necessarily lead to a higher risk of injury in this dataset.

#### 3.2.2. Multivariate Logistic Regression

**Figure 1:**
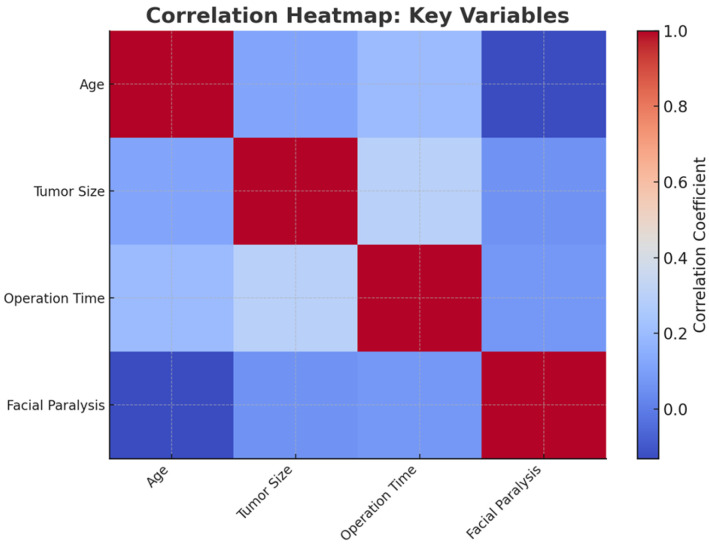
Correlation heatmap: This heatmap visualizes the correlation coefficients between key variables related to facial nerve injury after parotidectomy. Positive correlations are shown in warm tones (red), and negative correlations in cool tones (blue). Variables such as age, tumor size, operation time, and facial injury are compared. A moderate correlation between tumor size and operation time is evident, while weaker correlations exist between other variables. This visualization highlights how various factors interact in the dataset.

Logistic regression analysis was conducted to identify independent predictors of injury. Confidence intervals (CIs) and *p*-values were calculated to assess the significance and precision of the estimated effects (Figure 1).

NIM usage: The use of NIM was independently associated with a reduced risk injury (*p* < 0.05, OR: 0.35, 95% CI: 0.25 to 0.50), indicating its protective effect. The OR for NIM usage suggests that patients undergoing surgery with NIM were less likely to develop facial paralysis or palsy.

Age: Increasing age was identified as a risk factor for developing paralysis or palsy (*p* < 0.05, OR: 1.15, 95% CI: 1.10 to 1.20). The OR for age indicates that risk increases with each additional year of age.

**Figure 2:**
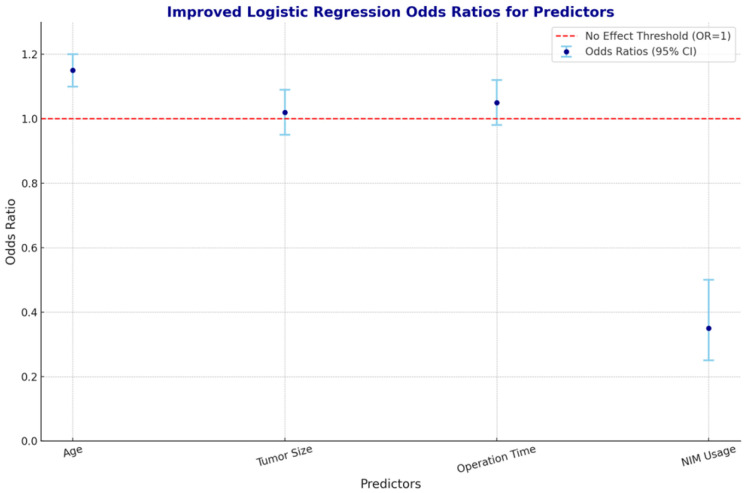
Logistic regression odds ratios for predictors: This figure illustrates the odds ratios (ORs) with 95% confidence intervals for key predictors of facial nerve injury following parotidectomy. Each point represents the odds ratio for a specific predictor variable. A red dashed line at OR = 1 marks the threshold of no effect. Variables with OR > 1 increase the likelihood of facial injury, while OR < 1 indicates a protective effect.

Tumor size (OR: 1.02, 95% CI: 0.95 to 1.09), operation time (OR: 1.05, 95% CI: 0.98 to 1.12), tumor site (OR: 0.85, 95% CI: 0.60 to 1.20), and histological type were not independently associated after adjusting for other factors (Figure 2).

#### 3.2.3. Exploration of Interactions

The potential interaction between tumor site and NIM usage was assessed using interaction terms in the logistic regression model. No statistically significant interaction was found, indicating that the protective effect of NIM is consistent across tumor locations, regardless of whether the tumor is in the superficial or inferior parotid lobe.

#### 3.2.4. Stratified Analysis

By tumor site: Tumors in the deep lobe showed a significantly higher incidence of nerve injury compared to the superficial lobe (9.5% vs. 3.2%, *p* = 0.035). Tumors in the inferior portion of the deep lobe demonstrated the highest risk, likely due to the proximity of these areas to primary branches of the facial nerve.

By histological type: The effect of NIM usage on facial nerve injury was also analyzed by histological subgroups. No statistically significant associations were found in pleomorphic adenoma (χ^2^ = 0.602, *p* = 0.438) or Warthin’s tumor (χ^2^ = 0.262, *p* = 0.609), indicating that the protective effect of NIM does not vary significantly by tumor histology.

## 4. Discussion

Facial nerve dysfunction is a well-recognized complication of parotid gland surgery, particularly in cases where the tumor’s location poses challenges due to the anatomical relationship with facial nerve branches. This study aimed to evaluate the incidence of facial nerve paralysis or palsy in patients undergoing surgery for benign parotid tumors, with and without the use of NIM, and to identify key factors influencing postoperative outcomes. Our findings were compared to several key studies in the literature, providing a comprehensive understanding of the role of surgical technique, tumor characteristics, and NIM monitoring in determining patient outcomes.

The study population had a higher proportion of females (52.9%) compared to males (47.1%), with a mean age of 61.3 years. This demographic is consistent with previous reports on benign parotid tumors; similarly, the mean tumor size of 3.1 cm aligns with findings from other studies focused on benign parotid neoplasms [1,7,10,12].

One of the primary findings of our study was the relatively low rate of permanent facial paralysis (4.6%) and palsy (0.6%), which aligns with other reports in the literature [7,10]. For instance, Grosheva et al. documented a 6% incidence of permanent facial nerve injury after parotidectomy for benign tumors [13]. However, Klintworth et al. reported a lower rate of 2% for permanent paralysis following extracapsular dissection, a less invasive technique that minimizes facial nerve exposure. This variation underscores the importance of surgical techniques in determining nerve outcomes, with less invasive approaches potentially reducing postoperative dysfunction, although they must be weighed against the risk of tumor recurrence.

Another critical observation was the significant role of tumor location. Tumors in the upper and deep lobes were associated with a higher incidence of both temporary and permanent facial nerve injuries, consistent with the anatomical proximity of the facial nerve branches in these regions [1,4,12,14]. These findings highlight the importance of preoperative planning and the selection of surgical approaches tailored to tumor location to minimize nerve injury risk.

Age emerged as a significant risk factor for postoperative facial nerve dysfunction, with the odds ratio increasing with each additional year of age. The higher likelihood of nerve injury in older patients may be attributed to age-related changes in nerve and tissue elasticity, vascular supply, and overall resilience. This finding suggests, as supported by other studies in the literature, that surgical strategies and postoperative care must be carefully tailored to older patients to mitigate these risks [15,16,17].

The use of NIM was a significant focus of this study. Our results demonstrated that NIM significantly reduced the incidence of permanent facial nerve damage, particularly in anatomically complex cases [12,18]. NIM provided real-time feedback during tumor resection, enabling more precise nerve handling and potentially contributing to improved outcomes. However, the literature remains divided on the utility of NIM, with some studies suggesting no significant benefit in reducing postoperative paralysis [19,20,21]. These discrepancies may be explained by differences in study design, patient populations, and the types of nerve monitoring used, as well as studies that included both benign and malignant tumors.

Tumor histology did not emerge as a significant predictor of nerve injury, in line with previous studies [1,5,12,13,22]. While histological subtypes such as pleomorphic adenoma and Warthin’s tumor are common in parotid surgery, factors like tumor size, location, and surgical dissection extent were more critical determinants of facial nerve outcomes. These findings suggest that histology may provide limited predictive value for nerve injury, reinforcing the importance of anatomical and procedural considerations during surgery. This is supported by Klintworth et al., who observed that benign tumors, such as pleomorphic adenomas and Warthin’s tumors, generally have a low recurrence rate and do not significantly affect facial nerve outcomes when proper surgical techniques are employed [4]. It must be remembered that facial nerve injury, such as palsy or paralysis, is a consequence of different factors starting from direct nerve transection without microvascular anastomosis, clamping, electrothermal damage, stretching, edema, trauma from aspiration or ischemia [10].

This study has several limitations that must be acknowledged: the retrospective design limits the ability to establish causal relationships and introduces potential selection bias. Additionally, as a single-center study, the findings may not be generalizable to broader populations. Although standardized criteria were used for data collection, variations in clinical documentation, particularly in subjective assessments of nerve function, could affect result reliability. Future multicenter, prospective studies with larger cohorts are essential to validate the protective role of NIM and to explore other risk factors, including comorbidities and surgeon expertise. Long-term follow-up studies are also needed to assess the recovery of facial nerve function over time and its impact on patient quality of life.

## 5. Conclusions

This study highlights the importance of tumor location, surgical technique, and the adjunctive use of intraoperative nerve monitoring (NIM) in minimizing facial nerve injury during parotid surgery. NIM was shown to provide a protective effect against permanent facial paralysis and palsy, particularly in anatomically complex cases, though careful surgical technique remains paramount.

Tumor location, especially in the deep and inferior lobes, emerged as a significant risk factor, while tumor histology, size, and operation time had no independent impact on nerve outcomes. Less invasive techniques, such as extracapsular dissection, offer potential advantages in reducing nerve dysfunction but require careful patient selection to balance recurrence risk. Even if NIM should be considered a standard practice during parotid surgery, as it provides real-time feedback that can help prevent nerve injury, it must not be considered a substitute for surgical anatomy knowledge and careful nerve dissection during the procedure [21].

## Data Availability

The data generated and analyzed during this study are not publicly available due to institutional and privacy policies but are available from the corresponding author upon reasonable request.

## Abbreviations

The following abbreviations are used in this manuscript:

ECD	Extracapsular enucleation
SP	Superficial parotidectomy
TP	Total parotidectomy
IONM	Intraoperative nerve monitoring
NIM	Nerve integrity monitor
SD	Standard Deviation
